# Sesame, an Underutilized Oil Seed Crop: Breeding Achievements and Future Challenges

**DOI:** 10.3390/plants13182662

**Published:** 2024-09-23

**Authors:** Saeed Rauf, Taiyyibah Basharat, Adane Gebeyehu, Mohammed Elsafy, Mahbubjon Rahmatov, Rodomiro Ortiz, Yalcin Kaya

**Affiliations:** 1 Department of Plant Breeding and Genetics, College of Agriculture, University of Sargodha, Sargodha 40100, Pakistan; saeedbreeder@hotmail.com (S.R.); taiyyibahbasharat@gmail.com (T.B.); 2 Department of Plant Breeding, Swedish University of Agricultural Sciences, P.O. Box 101, 23053 Lomma, Sweden; adane.gebeyehu.demissie@slu.se (A.G.); mohammed.elsafy@slu.se (M.E.); makhbubdzhon.rahmatov@slu.se (M.R.); 3 Department of Genetic and Bioengineering, Engineering Faculty, Trakya University, Edirne 22030, Turkey; yalcinkaya@trakya.edu.tr

**Keywords:** abiotic stress, capsules, CRISPR/Cas9, genomics, SNPs, yield

## Abstract

Sesame seeds and their edible oil are highly nutritious and rich in mono- and polyunsaturated fatty acids. Bioactive compounds such as sterols, tocopherols, and sesamol provide significant medicinal benefits. The high oil content (50%) and favorable mono- and polyunsaturated fatty acid balance, as well as resilience to water stress, make sesame a promising candidate crop for global agricultural expansion. However, sesame production faces challenges such as low yields, poor response to agricultural inputs, and losses due to capsule dehiscence. To enhance yield, traits like determinate growth, dwarfism, a high harvest index, non-shattering capsules, disease resistance, and photoperiod sensitivity are needed. These traits can be achieved through variation or induced mutation breeding. Crossbreeding methods often result in unwanted genetic changes. The gene editing CRISPR/Cas9 technology has the potential to suppress detrimental alleles and improve the fatty acid profile by inhibiting polyunsaturated fatty acid biosynthesis. Even though sesame is an orphan crop, it has entered the genomic era, with available sequences assisting molecular breeding efforts. This progress aids in associating single-nucleotide polymorphisms (SNPs) and simple sequence repeats (SSR) with key economic traits, as well as identifying genes related to adaptability, oil production, fatty acid synthesis, and photosynthesis. Additionally, transcriptomic research can reveal genes involved in abiotic stress responses and adaptation to diverse climates. The mapping of quantitative trait loci (QTL) can identify loci linked to key traits such as capsule size, seed count per capsule, and capsule number per plant. This article reviews recent advances in sesame breeding, discusses ongoing challenges, and explores potential strategies for future improvement. Hence, integrating advanced genomic tools and breeding strategies provides promising ways to enhance sesame production to meet global demands.

## 1. Introduction

Sesame (*Sesamum indicum* L.) is among the oldest oilseed crops known to humanity and is often referred to as the “queen of oilseed crops”. It is a diploid species (2n = 2x = 26 chromosomes), although variability in chromosome numbers has been identified in other species within the genus *Sesamum* [1]. Species such as *S. calycinum*, *S. latifolium*, and *S. angolense* are also diploids (2n = 2x) but with 32 chromosomes [1].

Sesame is an oilseed crop containing more than 50% edible oil and a balanced ratio of unsaturated and saturated fatty acids. Seed is also a rich source of functional molecules such as tocopherols, sterols, and sesamol. These molecules are not only beneficial for human health, but they also improve the shelf life of the oil due to their antioxidative properties. However, sesame has low yield potential when compared with other oilseed crops such as canola or sunflower, and little breeding efforts were made to improve yield potential, the introgression of host plant resistance to pathogens and pests, and shattering resistance [2]. The incorporation of genes for these characteristics may help to improve the harvestable yield of the sesame crop.

Sesame is grown in an area of 12.81 million ha, and 6.55 million metric tons were produced worldwide in 2022 [3,4]. Evidence suggests that the crop originated in Africa, where significant genetic diversity includes many wild relatives that are endemic to the various regions of this continent. Hence, Africa is considered its center of diversity. Sudan is the largest producer of sesame seed in the world followed by Myanmar and India [3,4].

Sesame is a self-pollinated, semi-erect plant that grows to a height of 0.6 to 1.2 m. Its ovate or lanceolate leaves vary, with basal leaves being tri-lobed and upper leaves irregular and serrated. As a drought-tolerant crop, sesame prefers well-drained soils with a pH of 5.5 to 6.8 and is suited to temperate tropical climates. The bell-shaped flowers have petal colors ranging from pale yellow to purple. However, sesame has a low yield potential and typically responds poorly to farm inputs, thus making it an unattractive option for growers compared to other oilseed crops. Continuous cultivation of sesame in the same field leads to declining yield, protein, and mineral content over time [5]. Additionally, the crop faces challenges such as shattering at maturity, which makes harvest difficult, and insect and disease infestations causing significant yield loss globally. Concrete efforts in breeding and genetics are needed to develop elite, high-yielding sesame cultivars with host plant resistance to insects and other pathogens. Current developments in breeding, genomics, and marker-assisted selection, therefore, highlight the importance of this crop.

## 2. Germplasm Resource Collection and Diversity Study in Sesame

### 2.1. Germplasm Resource Collection and Conservation

The primary breeding objectives for sesame are to increase seed yield, improve plant morphology, enhance tolerance to biotic and abiotic stresses, develop indehiscent capsules, and improve oil quality. These varied breeding goals highlight the critical importance of germplasm collection and conservation. A robust and diverse genetic repository is essential for breeders, providing access to a wide range of traits and alleles needed to develop improved cultivars that meet evolving agricultural challenges and consumer demands. Maintaining a comprehensive germplasm collection ensures the preservation of genetic diversity, which is crucial for the long-term sustainability and advancement of sesame breeding programs. This genetic resource is the foundation for future crop improvement innovations, climate change adaptation, and food security. Hence, germplasm collection and conservation is a continuous process to protect wild resources for sustainable agriculture. It has been thought that sesame originated from the wild populations native to Pakistan and Afghanistan. Alternative to this hypothesis, several wild species that are native to Africa could be also ancestors of cultivated sesame. Genetic research may be required to establish a relationship between modern cultivars and their parental population.

*Sesamum indicum* germplasm is conserved, inter alia, at the Brazilian Gene Bank (BGB, Brazil), International Crops Research Institute for the Semi-Arid Tropics (ICRISAT, India), the National Bureau of Plant Genetic Resources (NBPGR, India), the National Institute of Agricultural Science and Technology (NIAST, Republic of Korea), the Nigerian National Gene Bank (NNGB), the United States Department of Agriculture—Agricultural Research Service (USDA-ARS, USA), and Vavilov Institute of Plant Industry (VIR, Russia). ICRISAT conserves 1302 accessions of sesame germplasm, including wild accessions and those collected from international germplasm exchange. Similarly, NBPGR conserves 5288 accessions of sesame germplasm [6], while 11,116 accessions are held at the USDA-ARS repository, 9221 accessions at VIR, 3317 accessions at NIAST, 1455 accessions at BGB, and 1039 accessions at NNGB [6]. Evaluation and characterization of these accessions for morphological, agronomical, and molecular traits have been carried out, resulting in the identification of unique genotypes with high yield, disease resistance, and quality traits. For example, the FGsesame (http://ncgr.ac.cn/SesameFG/, accessed on 9 May 2024) database includes core collections of 705 sesame accessions from 29 countries, each identified based on origin, ecotype, sequencing coverage, and group information [7].

Germplasm resources are also crucial for ecosystem sustainability and nutritional security. The utilization of these resources in breeding programs, therefore, can lead to the development of improved sesame cultivars. The wild relatives of sesame contain unique genes that are not found in the cultigen pool [8]. It is, therefore, important to protect them from over-exploitation by local communities for culinary, medicinal, and cosmetic uses to prevent their extinction and maintain ecosystem balance.

### 2.2. Diversity Studies in Sesame

Research has demonstrated high genetic diversity among wild sesame relatives and cultivated germplasm from various regions. Wild relatives of sesame offer valuable traits for breeding programs, such as cytoplasmic male sterility, heat tolerance, disease resistance, and fatty acid modification. For instance, *S. radiatum* has more linolenic acid than other species [9]. The wild population of *S. orientale* closely resembles cultivated types (*S. indicum*) in both morphology and cytogenetics. Both species are diploid with 26 chromosomes and produce fertile hybrids under spontaneous or artificial reciprocal hybridization. CpDNA-based fingerprinting also supports a close relationship between *S. orientale* and *S. indicum* [10]. Its origin is still uncertain but some authors suggest it originates from the African population of *S. latifolium* as it is widely cultivated and found growing wild in tropical regions [11].

Crosses between wild and cultivated species have successfully introgressed desirable traits, such as charcoal rot resistance [12]. However, crosses between *S. indicum* and *S. latifolium* were largely unsuccessful, producing only a few seeds due to chromosomal number and structural differences (*S. latifolium* has 2n = 32 chromosomes). Both species also show qualitative differences, such as the absence of sesamolin in *S. latifolium*, thereby indicating both cytological and morphological differentiation between the species.

Molecular, biochemical, and morphological markers have been used to assess diversity among sesame accessions. Isozyme analysis and morphological trait comparisons have differentiated wild and cultivated species [1,13]. The presence of polymorphic co-dominant cathodic and anodic peroxidase isozymes, Co-Px-2 and An-Px-2, is unique to wild sesame germplasm. Additionally, the *Sh-1* marker distinguishes *S. indicum* from wild *S. mulayanum*. Similarly, the *Sh-1* marker can distinguish *S. orientale* from other wild accessions, confirming genetic diversity in wild sesame.

The development of molecular markers, such as simple sequence repeats (SSR) and single-nucleotide polymorphisms (SNP), has also been instrumental in the genetic diversity analysis and mapping of quantitative trait loci (QTL) in sesame [14]. SSR-based research has shown genetic diversity indices and divergence among Indian accessions [15]. Expressed sequence tag (EST) databases were exploited for the development and characterization of gene-derived SSR markers using the computer-based software MISA version 1.1.2, while SNPs were detected and visualized using the QualitySNPng software tool (http://www.bioinformatics.nl/QualitySNPng, accessed on 9 May 2024). SNP variants were identified through a genome comparison of sesame. The sesame genome survey identified SSR markers that could be exploited to determine the genetic diversity and QTL mapping of economic traits. There were 23,438 SSR (five repeats) markers, with dinucleotides being the most prevalent repeat pattern (84.24%), followed by trinucleotides (13.53%), tetranucleotides (1.65%), pentanucleotides (0.3%), and hexanucleotides (0.28%) [14]. Identifying microsatellites in the sesame genome led to the development of 218 polymorphic SSR markers for screening sesame germplasm for genetic diversity analysis [14]. The sesame cultivar “Sweeta” genome sequences were obtained through Illumina paired-end sequencing and 454 shotgun sequence technologies. Reports on the genetic diversity within diverse global accessions or native landraces are given in Table 1. Improved assembly and annotation of the genome were performed by high-quality sesame genome assembly using PacBio and Hi-C technology, improving continuity and chromosome anchoring. The new assembly identifies 24,345 protein-coding genes and reveals ancient whole-genome duplication events. These advancements will support future genetic research and breeding efforts in sesame [16]. The GINMicrosatDb database of microsatellites was developed, containing five sets of primer pairs for each microsatellite locus [17]. The database also included GC content, melting point, and flanking sequences [17].

**Figure 1:**
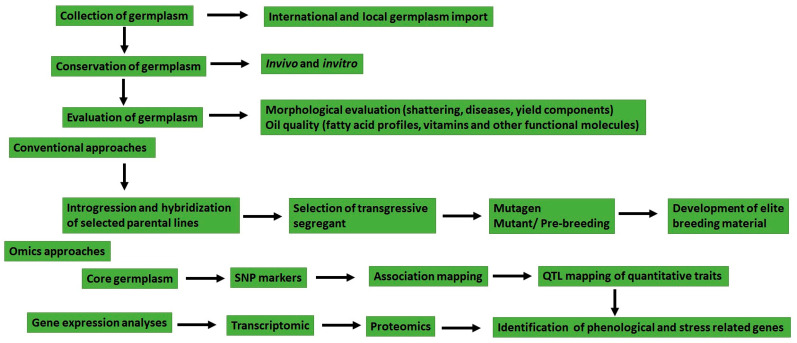
Flow chart of activities related to sesame breeding and germplasm conservation.

Transcriptomic profiling of sesame genes revealed 57 MADS-box genes across 14 linkage groups that are essential for sesame development and growth [7]. Structural analysis and functional differences among MADS-box genes showed similarities and differences with model species *Arabidopsis thaliana* and *Solanum lycopersicum* [7]. The MIKCC-type MADS-box genes are also shown related to sesame flower initiation and seed development [7]. Transcriptomic analysis of interspecific hybrids has also identified genes related to disease resistance [18]. Studies of 4.4 Mbps related to fatty acid metabolism show genetic diversity loss during the domestication of sesame [19]. Expression profiles of traits such as capsule, seed leaf, and root development in cultivar “Zhongzhi13”, as well as the transcriptomic profile of genes related to waterlogging tolerance in “ZZM2541” and oil content in high-oil-yielding cultivar “Zhongfengzhi1”, were investigated [7]. Candidate genes related to seed yield, lipid metabolism, phenological traits, or disease resistance are available in the database [7]. Hence, wild and cultivated accessions should be further evaluated and characterized for morphological, agronomical, and molecular traits and consequently used as parental material to develop pre-breeding lines or improved sesame genotypes with high yield, disease resistance, and quality traits. A comprehensive summary illustrating the concepts discussed in the text on sesame breeding and conservation is illustrated in Figure 1 below.

**Table 1:** Genetic diversity estimates in diverse sesame germplasm across global and local accessions.

Germplasm	Origin	Markers	Diversity	Reference
183 accessions: 101 Chinese accessions, 62 landraces, and 20 exotic accessions	China	25 simple sequence repeat (SSR) markers	0.27–0.65 Shanon information index Moderate	[20]
2496 sesame accessions: 17 countries and 26 states of India	India	43 SSR markers	1.14–2.07 Shanon information index	[21]
35 sesame landraces	Greece, Bulgaria, Turkey, Iraq, Yeman, Tajikistan, Korea	7 expressed sequence tag (EST)-SSR markers	0.27–0.35 Shanon information index 0.15–0.21 Diversity index	[22]
6 sesame cultivars	Saudi Arabia	20 inter simple sequence repeat (ISSR) and 25 SCoT primers	Inter simple sequence repeat (ISSR) vs. start codon targeted (SCoT) polymorphisms according to polymorphism information content (PIC) 65% vs. 78% 0.26–0.31 Shanon information index	[22]
100 sesame accessions	Ethiopia	27 SSR markers	Mean gene diversity 0.30	[23]
300 sesame accessions	Africa and Asia	5065 silico diversity array technology (DArT) and 5821 single-nucleotide polymorphism (SNP) markers	Average diversity 0.14 Asia (0.17) vs. Africa (0.14) Africa (0.21) vs. North Africa (0.23)	[24]
501 accessions	Africa, Europe, America, Asia	24,735 SNP markers	High degree of differentiation (0.27)	[25]
2496 sesame accessions	National Genebank of ICAR-National Bureau of Plant Genetic Resources	64,910 SNP markers	384 accession (15%) represent maximum diversity. Captured 60% of the SNP diversity	[26]

## 3. Oil Content and Fatty Acid Composition Analysis in Sesame Germplasm

Oil content in sesame accessions varies depending on genotype and environmental interaction, ranging from 45% to 53% among accessions from Turkey and 11 other countries [27]. The predominant fatty acids in sesame oil are oleic acid (36–43%), linoleic acid (39–46%), and palmitic acid (8–10%) [27]. Sesame oil is rich in unsaturated fatty acids, making it suitable for culinary and industrial applications (Table 2). A recent study utilized genome-wide association analysis (GWAS) and transcriptome analysis to identify novel loci and regulatory genes involved in fatty acid biosynthesis [28]. The study discovered that specific gene clusters, including *FAD2*, *LOC10515945*, *LOC105161564*, and *LOC105162196*, play crucial roles in regulating unsaturated fatty acid biosynthesis. Additionally, a regulatory co-expression network was constructed based on differentially expressed genes, aiding in understanding the transcriptional and translational regulation of oil biosynthesis in sesame seeds. These findings provide a molecular platform for improving the quality and yield of sesame oil, potentially benefiting breeding programs aimed at developing sesame cultivars with higher levels of unsaturated fatty acids.

Major lignans in sesame seeds include sesamin and sesamolin, with higher concentrations found in white-seeded cultivars [29]. Sesame seeds are also a rich source of minerals, with calcium being the predominant cation, followed by potassium and magnesium [30]. The seeds contain various phenolic compounds that impart antioxidant properties, which may help prevent disease [31].

**Table 2:** Natural variation for oil content (%) and fatty acid composition (%) in sesame germplasm.

Accession	Oil Content	Protein	Fatty Acids						Reference
			16:0	18:0	18:1	18:2	18.3	22:1	
22 cultivars, 4 landraces of *S. mulayanum*, and 7 accessions of 4 wild species	–	–	9–15	–	37–52	30–52	–	0–8	[19]
4 Pakistani sesame varieties	50–54	19–23	3–19	5–22	10	5–13	–	16	[32]
Market sample	48		5	13	43	36	–	–	[33]
Brown non-roasted vs. roasted sesame	49 vs. 51	–	5.6 vs. 7.1	5.3 vs. 6.8	40.3 vs. 58.7	46.1 vs. 25	0.4 vs. 0.2	–	[34]
103 sesame landraces	41–63	–	8–10	–	29–41	41–49	–	–	[35]
Collection of 12 countries	45–53	–	5–6	8–10	36–44	39–46	0.3–04		[36]
6 cultivated (C) 3 wild spp. (W)	53–55 (C) 54–59 (W)	–	4–9 (C) 4–8 (W)	4.5–10 (C) 4–5 (W)	33–38 (C) 35–37 (W)	42–52 (C) 43–46 (W)	4–9.5 (C) 5–10 (W)	–	[9]
Indian sesame cultivar	42–54	16–27	8–12	4–8	41.5–50	32–43	0.2–0.35	–	[36]

## 4. Fatty Acid Biosynthesis and Metabolism in Sesame

Fatty acids in sesame are synthesized de novo within the chloroplasts and can be esterified with glycerol to form triglycerides, which are stored as oil bodies in plant tissues. The fatty acid composition dictates the end use of the oil, with higher polyunsaturated fatty acids being unsuitable for deep frying due to oxidation [37]. Efforts to modify fatty acids in crops like Brassica, sunflower, and soybean through genetic techniques have aimed to improve oxidative stability and nutritional value [38,39,40]. Fatty acid accumulation starts 21–33 days after flowering. There are 38 different types of fatty acids in sesame, which were detected through gas and liquid chromatography [41]. Candidate genes for the oil and fatty acids can be identified through RNA profiling. RNA profiling in sesame showed the role of various candidate genes, e.g., *CCase*, *FAD2*, *DGAT*, *G3PDH*, *PEPCase*, *WRI1,* and *WRI1-like* genes [42]. Efforts to enhance ω-3 fatty acid content could address deficiencies in human diets, although such oils may be unsuitable for cooking due to rapid oxidation [38].

**Figure 2:**
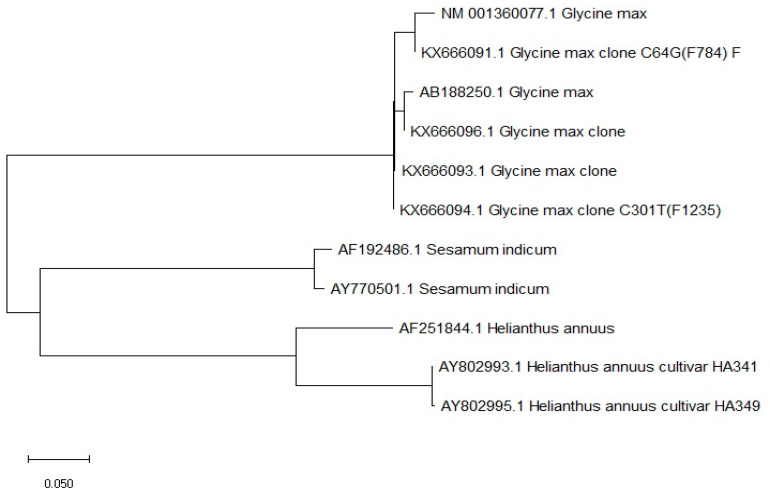
Cluster and phylogenetic analysis among various accessions of *Glycine max*, *Sesamum indicum*, and *Helianthus annuus* based on the *FAD2* gene. Phylogenetic analysis was based on the sequence homology of FAD2 transcripts downloaded from gene bank https://www.ncbi.nlm.nih.gov/genbank/, accessed on 9 May 2024 and analyzed through MEGA X software.

To increase stearic acid concentration, it is crucial to enhance the activity of acyl-ACP thioesterase and suppress the *SAD1* gene [43,44]. Blocking ω-6 fatty acid desaturase activity can improve oleic acid concentration in sesame oil [45]. The sequence homology of FAD2 transcripts obtained from wild and cultivated species showed divergence and SNP among both groups of germplasm [46]. Polymorphism within nucleotides led to the change of amino acids in the active site of the enzymes, leading to the differential ability of enzymes to catalyze linoleic acid contents within wild and cultivated germplasm [46]. It has been shown that abscisic acid induces seed-specific expression of the *SeFAD2* gene, which is controlled by introns [47]. *FAD2* gene expression may be blocked through anti-sense technology that helps to raise the concentration of oleic acid. FAD2 complete transcripts in various crop species such as soybean, sunflower, and sesame were obtained through an online gene bank database. Sequences were downloaded, and their relative sequence homology was compared on MEGA X software version 11.0.10 (Figure 2). It showed that species could be characterized based on the sequence homology for *FAD2* genes showing their independent orthologous evolution. High genetic differentiation within the *FAD2* gene between species may lead to various versions of the oleate desaturase enzyme. Soybean accessions showed the highest sequence homology, showing low genetic differentiation within species while sunflower had the highest genetic differentiation between their accessions (Figure 2).

## 5. Sesame Wilting Disease

Sudden wilting in sesame is a common production constraint in hot and dry regions of the world. Initially, it was known that the disease was caused by *Fusarium oxysporum* [48]. However, it has been observed that sudden wilting syndrome was caused by cumulative biotic and abiotic factors. In Pakistan, sudden wilting syndrome was one of the major production constraints due to the hot climate and caused 50–100% yield loss across the belt of central Punjab, Pakistan. Sudden wilting is observed in hot climates (40 °C) after delayed irrigation, and plants experience wilting within 24 h. The infected plants had *Fusarium oxysporium* and *Macrophomina phaseolina* [49]. However, many of the samples did not carry any of the two pathogens, but abiotic factors such as heat and drought often lead to the sesame wilt. The incidence of wilting increased with delayed irrigation [49].

**Figure 3:**
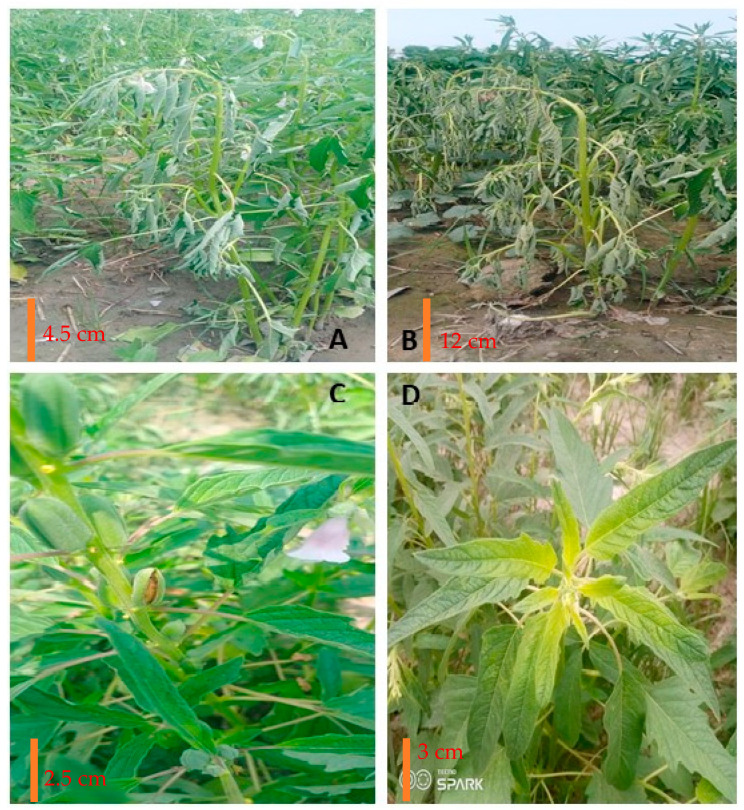

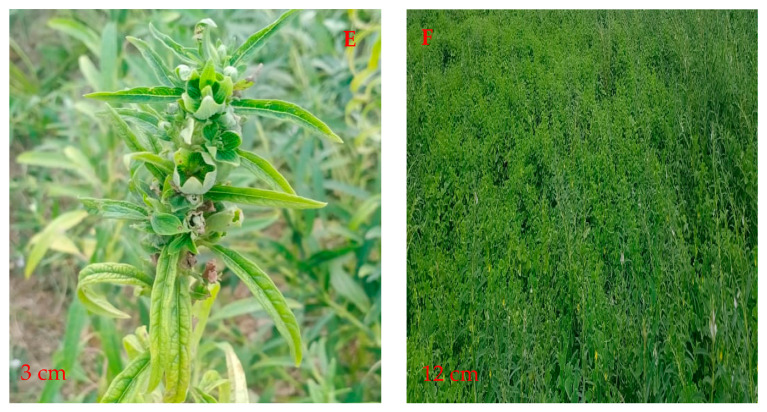
Sesame wilting syndrome after delayed irrigation (**A**), under high temperature (**B**), high-temperature effects on sesame causing stunted growth, flower shedding, and mal capsule formation (**C**), and sesame pod rotting in humid conditions (**D**). Phyllody disease causing the conversion of the capsule into a flower-like structure (**E**) and the sesame crop affected by the weed infestation (*Cucumis callosus*) causing the suppression of pod formation during the reproductive phase (**F**).

A significant increase in the browning of the stem in the wilted plants of sesame was observed after 5–6 days of rain (20 mm) in heavy soils of Shahkot, Pakistan, indicative of charcoal rot infestation after wilting (Figure 3). Resistance breeding may be required to cope with the sudden wilting in the sesame. It may require the screening of breeding material and the development of cultivars that may survive hypoxia conditions within roots after heavy rainfall or irrigation [50]. Moreover, the introgression of charcoal or root rot disease resistance may further reduce the chances of fungal infestation after the wilting. Combining ability analyses of the sesame breeding lines were performed to determine the breeding value for charcoal rot resistance. Selectable variation for charcoal rot resistance was low to high, and there were significant environmental and dominance effects associated with the charcoal rot resistance which indicates that recurrent selection and genotypic selection will be practiced to improve the charcoal rot resistance [51]. The sesame genetic map was developed from 424 new polymorphic markers and 548 recombinant inbred lines [52]. These markers detected 14 QTLs which explained 3–14% of the total genetic variation contributing to the charcoal rot resistance [20]. The *qCRR12.2* contributed the highest to charcoal rot resistance in all four environments, and *qCRR8.2* and *qCRR8.3* were environment specific. These QTLs were used to predict the disease-resistance genes. Genome-wide association research may be required to tag genes for resistance breeding.

## 6. Abiotic Stress Tolerance in Sesame

Sesame is a drought-tolerant crop with nitrogen recovery ability, making it suitable for arid climates with both low rainfall and low soil fertility [53]. Climate change, characterized by increasing CO_2_ levels and temperature fluctuations, poses significant challenges for sesame production. While sesame thrives in high temperatures, it experiences reproductive failures when temperatures exceed 33 °C [53]. Developing new cultivars with enhanced drought and heat tolerance, combined with high yield potential, is essential for sesame cultivation under changing climate conditions [54].

Selection for high yield potential and stability has been effectively carried out through the QTL mapping of economically important traits linked with yield, resistance against stresses, and stability (Table 3). Molecular markers and QTL mapping have been used to select high-yielding and stress-resistant cultivars [55]. Transcriptomic analysis of drought-tolerant sesame accessions has identified genes related to osmotic stress [56]. The root profile of drought-tolerant sesame accessions showed differential expression of genes related to catalytic activity, ion binding, and transferase activity compared to susceptible accessions [56]. This study, therefore, helps to identify the genes related to abiotic stresses such as osmotic stress.

Sesame is a short-day plant, and flowering is initiated in response to day length and temperature, with day length having a significant effect on sesame flowering time [65]. A gene named *CONSTANS (CO)* promotes flowering time in sesame [65]. It shows similarities to the zinc finger transcription factors of *Arabidopsis* and dictates the vegetative growth period of sesame. Understanding the genetic regulation of flowering and maturity can help develop cultivars suited to specific environments [57]. Flowering time may be a multigenic trait and numerous loci may show genetic interactions. In this regard, generation means analysis of flowering time in sesame indicated that two genes have additive, dominant, and epistatic effects [65]. It also showed a negative relationship with other morphological, growth, and yield-related traits [66]. Accessions with later flowering had a small reproductive period. 

Gene regulation of flowering initiation in sesame could help to understand the mechanism underlying flowering and maturity under a specific set of environments. The gene *CO* has two homologs (*SiCOL1* and *SiCOL2*. *SICOL2*) lacking the B-box motif. It delays flowering compared to genotypes with the *SiCOL1* sequence. *SiCOL1* is similar to the *CO* gene, showing high expression in leaves before flowering and diurnal rhythmic expression in response to day length. It has 16 haplotypes within Asian genotypes, and the mutant type does not respond to day length, indicating it is non-functional or photoperiod-insensitive. Genotypes having a mutant allele of *SiCOL1* initiated early flowering under long days [57]. Hence, genome sequencing and annotation may help identify differentially expressed genes and understand the adaptability of sesame genotypes to a particular environment.

## 7. Inducing New Variability within Sesame Germplasm

### 7.1. Mutation Breeding

Sesame crop has defective phenology, with pod shattering, susceptibility to diseases, poor response to farm inputs, and insect infestation. To overcome these problems and to obtain new genetic variability within elite germplasm, a mutation breeding program was initiated in various parts of the world. New genetic variation was induced through the use of various physical and chemical mutagens (Table 4). At least 25 mutants have been released for general cultivation with improved agronomic and stress resistance under various mutation breeding programs in eight countries [67]. A dose of chemical mutagen of 0.5% or 1% ethyl methane sulfonate (EMS) has been recommended to induce novel genetic variability, which generated novel capsule-bearing traits such as tetra- or tri-capsule per leaf axil, determinate growth habit, and seed color [68]. The optimum lethal dose for gamma radiation was 389–417 Gy [69]. Selection within the mutant population and progeny evaluation of advanced mutant lines (M5) led to the selection of candidate lines SM-06, SM-07, and SM-04. These candidate lines were superior in terms of early maturity, and higher yield components such as capsule plant-1 when compared with standard checks [70]. Mutation breeding had significant effects on traits related to earliness and days to 50% flowering. These traits also showed significant variability within mutant lines [71]. In another study, advanced mutants (M3) were screened for high oleic acid content (70%). Six mutants from M3 were advanced to M6–M7 which showed oleic acid contents of about 70% when compared with control lines [42]. The genetic basis of high oleic acid content (%) was also investigated. A nucleotide mutation within the *FAD2* gene led to the substitution of amino acid in oleate desaturase, thus inducing an increase in the oleic acid contents [46].

Mutation breeding programs generally had random effects on the genome, and generated mutants may contain several mutations within non-targeted traits. Traditional mutants are used as pre-breeding material to transfer genetic variation into elite germplasm. Contrastingly, CRISPR/Cas9 could be used for targeted genome modifications in crops. The mutants released through CRISPR/Cas9 may have the least detrimental effects on yield and related traits. In this era of genomics, new genes for photoperiod sensitivity, fatty acids, shattering, growth habit, pathogen, and insect resistance will be constantly identified. CRISPR/Cas9 may be used as a tool to downregulate undesirable genes. The downregulation of genes related to fatty acids may help to develop high-steric- or –oleic-acid mutants, while genes related to the shattering problem may be knocked out to develop shattering-resistant mutants which helps to minimize yield losses and improve seed quality as well [72]. Sesame-specific adaptability may be improved by downregulating genes related to photoperiod. Photoperiod insensitivity helps to initiate flowering under long day lengths [73]. Two sgRNA were designed in an attempt to downregulate the two cytochrome genes (*CYP81Q1* and *CYP92B14*) that are involved in the biosynthesis of sesamin and sesamolin [74]. Sequence data showed a mutation frequency of 90.63 and 93.33% in *CYP81Q1* and *CYP92B14*, respectively [75]. However, there were no off-target mutations identified in the study. The biosynthesis of sesamin and sesamolin was significantly disrupted in the transformed roots, which also confirmed the key functions of the genes in lignan biosynthesis [75].

**Table 4:** Exposure of sesame for the establishment of a mutant population.

Mutagen	Established Population	Novel Variation	Reference
Sodium azide	M2	Glabrous stem	[76]
Sodium azide (15 mM)	M4 and M5	Early maturity, yield advantage of 21% during the Kharif season	[77]
600 Gy Co	M4	Salinity tolerance, number of capsules, 1000 seed mass	[78]
300 Gy gamma rays	416 M3 mutant	P 97-1 *Phytophthora nicotianae* resistance	[79]
40 KR gamma rays 1.5 mM	M2, M3	Early maturity, no. of capsules, seed yield plant^−1^	[80]
-	M2	Tetra-carpellate per leaf, higher number of seed capsules^−1^	[81]
40 mL 0.8% ethyl methane sulfonate (EMS) in 100 mM phosphate buffer	M3	M5 generation was subjected to screening, and plants with high oleic acid contents (75%) were selected	[82]
0.5% EMS Sorenson phosphate for 24 h at 4 °C	M2	A significant change in both qualitative and quantitative traits when 14 Ethiopian sesame genotypes were exposed to mutagen	[83]
250 Gy gamma radiation	M0	Germination was reduced by 50%	[84]

### 7.2. Development of Semi-Dwarf Plants and Various Mutant Types

Mutation breeding led to the evolution of a semi-dwarf sesame genotype (*dw607*). Semi-dwarf genotypes were characterized by reduced intermodal length, higher stem lodging resistance, and higher thousand seeds weight (TSW) due to larger seeds [85]. Molecular analysis of 824 accessions showed that *Sidwf1* affects plant height. These 824 accessions had 58 different genomic variants for *Sidwf1*, which has an SNP mutation of nucleotide substitution from C to T, thereby causing an amino acid change from proline to serine [85].

An EMS-induced sesame mutant (*sc1*) with few seed capsules and short capsules was due to a mutation of the *SICS1* allele [7]. Sequencing of loci *SICS1* showed a point mutation at intron 5 and exon 6 junctions which induced a nonsense mutation due to a frameshift of the reading frame. The overall effect was a defective protein [7]. The indeterminate growth habit in sesame is dominant over the determinate type [86]. Multiple allelism for determinate growth habit *dt1* and *dt2* was noticed in crosses between two growth types [70]. Long and dense capsules were dominant over short and sparse capsules when two types were crossed, and the F_1_ hybrid was phenotypically characterized. These two types showed a segregating ratio of 12:3:1, which indicated that the two genes controlling the long and dense capsules had epistasis interactions [87]. In another study, the *yl1* mutant was phenotypically characterized. The yellow-leaf dwarf mutant had lower carotenoid and chlorophyll contents than wild types. The *yl1* mutant also had a longer growth duration with a slower photosynthesis rate, later flower initiation, and shorter plant height than the wild type [88].

### 7.3. Indehiscence Capsule

Capsule shattering is a major production constraint in sesame and leads to 50% yield loss [72]. To reduce yield loss due to capsule shattering, farmers tend to harvest manually with partial maturity. Capsule dehiscence is a dominant trait in sesame, and crossing the indehiscent parent with the dehiscent parent results in an indehiscent F_1_ hybrid [89]. Moreover, a Mendelian ratio (3:1) was observed in the F_2_ generation where a single recessive allele was controlling the indehiscent capsule [90]. The identified allele for shattering resistance was *id Sindi0765000.* However, it has several pleiotropic effects and induces undesirable effects on plant phenotype. Generation means analysis obtained by crossing capsule-shattering-resistant and -susceptible parents also confirmed this finding [90]. Anatomical analysis of shattering-resistant mutant 12M07 showed a parenchyma cell was loosely arranged but adhered to the seed coat [91]. Marker-assisted selection has been proposed, and gene-controlling shattering or non-shattering types were mapped onto SNP marker S8_5062843 (78.9 cM) near the distal end of LG8 (chromosome 8) [91]. Sequence analysis indicated a frameshift mutation in the non-shattering alleles, which caused a shift in the splicing and introduced SNP variation compared with the shattering alleles [91]. In another study, association mapping showed that SNP SV12002 in the 5′ upstream region of gene *Sindi0765000* in chromosome 3 was closely associated with shattering resistance [92]. To facilitate the selection of non-shattering types, cleaved amplified polymorphic sequence (CAPS) markers were developed [65]. The shattering trait was mapped by 782 SNP markers, and shattering resistance was mapped on the distal part of LG8 in the population generated from Muganli-57 and PI 599446 [93]. A candidate gene for the shattering trait was identified that encodes a protein of 440 amino acids [93]. CRISPR/Cas9-based knockout within the candidate caused shattering resistance and leaf curling [93].

### 7.4. Male Sterility and Hybrid Vigor

Hybrid breeding provides another possibility for improving yield. Breeding lines with superior specific combining ability tend to show dominance and may also have shown superiority in yield and related traits. Economical heterosis ranged between 9.5 and 32.7%, as estimated in 1636 hybrid offspring [94]. Reliable genetic male sterility is a prerequisite for hybrid seed production. Genetic male sterility, including nuclear or cytoplasmic male sterility, may be induced through mutations. A list of various nuclear alleles identified or induced in sesame is given in Table 5.

Cytoplasmic male sterility sources were also discovered after interspecific hybridization. *S. malabaricum* was identified as a donor species for the sources of cytoplasmic male sterility [99]. Nuclear male sterility is controlled by two homozygous recessive alleles, which may be maintained in heterozygous conditions and upon self-pollination. The obtained progeny was subjected to the selection of male sterile plants through visual markers at the seedling stage [84]. Wrinkled leaves were used as a marker to differentiate male sterile plants [84]. Nuclear male sterility could also be induced spontaneously. For instance, recessive male sterility was identified in the Chinese cultivar “Zhuzhi”. This male sterility was transferred and maintained through sib mating and crossing with male fertile plants [100]. This source of nuclear male sterility was “D248A” [97]. Male sterility source “D248 A” had rudimentary small green anthers that were sterile and did not contain pollen. Abnormality in the pollen mother cells led to the anucleated microspores with less cytoplasm [97]. These microspore mother cells were not able to undergo normal meiotic division [97]. The segregation ratio of nuclear male sterility was 1:3 in the F_2_ generation. A dominant source of the genetic male sterility allele was also found in sesame [96]. This type of male sterility was easier to maintain than recessive genetic male sterility [97].

Male sterility may be restored through chemical agents which provide ease for the maintenance of genetic male sterility in sesame [101]. Genotype CRA-02 had 33% of male fertility when sprayed at bud initiation stages [101]. Male sterility and leaf curling were induced by the putative region at chromosome 4, which was identified through bulk segregant analyses followed by next-generation tools [93]. Region association with male sterility was further narrowed down through MAS by SSR and InDel markers in the backcross population BC_1_. Gene expression analyses showed suppression of four genes associated with sterile pollen and one gene during leaf development [92]. The gene cell wall invertase, *CWINV1*, was suppressed in male sterile plants, which were supposed to supply the carbohydrates to the developing pollen [102]. Cloning research showed that gene *Sicwinv1* associated with male sterility in sesame accumulates during the tetrad stage in the tapetum cells of the anther [102].

## 8. Genetics of Economic Traits

Yield is a complex trait, which is significantly affected by numerous loci that may interact with each other and with the environment or crop management [103]. QTL for yield components has been mapped on various linkage groups. In a study, major QTLs for seed size and seed coat color on LG04 and LG11 were mapped. There were about 155 candidate genes associated with seed coat and size in these linkage groups [103]. The genome-wide association study (GWAS) approach enabled the mapping of QTL in sesame with precision due to high-density markers within the genome. This GWAS approach has been instrumental in uncovering the genetic architecture of oil content and fatty acid composition in sesame seeds [55].

Whole-genome sequencing allowed high-density markers such as SNP or SSR to map quantitative traits of sesame [103,104]. For instance, a study in sesame used 2159 SNP markers on 13 linkage groups with a marker distance of about 0.99 cM [103]. About 83,135 non-redundant SSR marker positions and motif sequences have been published and obtained from 151 published genomic sequences [104]. It enabled the mapping of “hotspots” within the sesame genome that were related to various economically important traits. In another study, association mapping with a high-density linkage map of 19,309 markers revealed 84 QTLs associated with yield components and seed mineral components [92]. Thirteen QTLs on 7 LG and 17 QTLs on 10 LG were linked with yield components which were mapped through 1230 markers in the recombinant inbred line (RIL) population [105]. High-throughput phenotyping technologies have been combined with SNP mapping to characterize labor-intensive traits with more precision and greater efficiency. For example, near-infrared spectroscopy (NIRS) has been recommended to determine several seed-related traits such as fatty acid and oil contents, which may provide a rapid alternative to other techniques such as gas chromatography or soxhlet apparatus, and it may facilitate the screening of large breeding populations [106].

A study comprising 3528 high-density specific locus amplified fragment (SLAF) markers having an average distance of 0.37 cM was used to map quantitative traits. SLAF makers mapped 46 significant QTLs for seven yield components across four environments [107]. Association mapping revealed 23 stable QTLs, which were detected in all environments. Selectable QTLs were mostly in the Chinese line “Yuzhi4” [108]. Furthermore, integrated multi-omics data from genomics, transcriptomics, and metabolomics, have provided a more comprehensive understanding of sesame’s molecular mechanisms underlying complex traits. This systems biology approach has revealed intricate gene regulatory networks involved in oil biosynthesis and stress responses [28].

Association genetics using a whole-genome re-sequencing strategy with 1354 markers over 12 chromosomes identified QTL related with water-stress-related traits such as root length and relative root length [108]. There was significant genetic variation for yield components such as capsule size, capsule number, and seed-size-related traits. A QTL qLS15-1 affecting the leaf size and development was mapped on LG15 [109]. Furthermore, this QTL (qLS15-1) was subjected to the sequencing of QTLs and their transcriptome analyses led to the identification of *SIN_1004875*, *SIN_1004882*, and *SIN_1004883 I* as candidate genes [109]. These QTLs were stable across environments, were in LG15, and affected leaf size and development [109]. Their sequencing and transcriptome analyses also confirmed these candidate genes [109].

Capsule number and length are important yield components in sesame and may dictate the yield potential of an accession. A study revealed two novel candidate genes (*SiLPT3* and *SiACS8c*) affecting capsule number and capsule length. In another study, a high-density linkage map of 20,294 SNP markers led to identifying major QTL on LG2 for flowering date and yield components [110]. A significant QTL was related to the candidate gene *SIN_1019016* on chromosome 10, gave *Phytophthora* blight resistance, and explained up to 13.34% of its phenotypic variation [111]. The advent of genome editing, particularly CRISPR-Cas9, has opened new avenues for targeted modification of sesame traits. While still in its early stages for sesame, this technology holds promise for precisely manipulating genes controlling key agronomic traits, thereby accelerating the breeding process [75].

Genomic selection (GS) is a transformative approach in plant breeding. It enhances significantly the efficiency of breeding programs through the incorporation of vast genomic data. Unlike traditional marker-assisted selection, which focuses narrowly on specific genes or QTL, GS leverages comprehensive whole-genome information. This methodology allows breeders to predict the breeding values of plants by analyzing numerous genetic markers that are distributed throughout the genome. As a result, GS is particularly advantageous for improving complex multigenic traits, such as yield, oil content, and host plant resistance to pathogens.

Recent research conducted by Sabag et al. [112] highlights the potential of GS in sesame breeding. Their study demonstrated that single-environment analyses yielded moderate to high genomic prediction accuracy for various agronomic traits. Importantly, the authors concluded that employing multi-environmental trial data could further elevate genomic prediction accuracy, thus enhancing the development of sesame cultivars better suited to semi-arid climates. This finding underscores the importance of environmental context in genomic selection, suggesting that integrating diverse growing conditions can lead to more robust predictive models. Despite promising developments, it is noteworthy that comprehensive research on genomic selection and prediction specifically within sesame has been limited. As demand for resilient and high-yielding sesame cultivars grows, the application of GS offers a valuable avenue for breeders. By increasing the accuracy of trait predictions and accelerating the breeding process, genomic selection holds the potential to significantly boost genetic gains in sesame, ensuring a more sustainable agricultural future. As research in this area continues to expand, the incorporation of genomic methods and tools will undoubtedly play a pivotal role in advancing global crop improvement efforts. Hence, whole-genome sequencing, genomic selection, genomic prediction, and GWAS pave the way for breeding sesame in the 21st century.

## 9. Prospects for Sesame Improvement

Prospects for sesame improvement lie in integrating crossbreeding with advanced methods such as marker-assisted selection, genomic selection, and gene editing. Recent advances in high-throughput phenotyping and genotyping have significantly accelerated sesame improvement. For example, unmanned aerial vehicles (UAVs) equipped with multispectral sensors now allow for the rapid assessment of large-scale field trials, thus enabling more efficient selection of superior genotypes that will expand the area of this crop for new germplasm with adaptability to diverse environmental conditions. On the other hand, sesame has low yield potential due to poor response to farm inputs. Sesame accessions having characteristics such as determinate growth, semi-dwarf stature, and lodging resistance are required to improve response to farm inputs and the same ideotype for mechanized harvesting. Moreover, accessions with indehiscence capsules should be bred to reduce yield loss during harvesting. Additionally, developing sesame cultivars with synchronized maturity is another critical breeding objective, as it would significantly improve the efficiency of mechanical harvesting and reduce post-harvest losses. Some of the work has already been conducted, and germplasm accessions with novel agronomic traits were developed through mutation breeding, selection, or hybridization. The application of CRISPR-Cas9 technology holds promise for targeted gene editing to develop high-yielding, pest-resistant, and climate-resilient sesame cultivars. For example, CRISPR-Cas9 could modify genes involved in lignin biosynthesis, potentially developing cultivars with improved content of oil and easier extraction. Furthermore, expanding transcriptomic and proteomic research will enhance our understanding of the regulatory mechanisms controlling important agronomic traits. Hence, integrating omics approaches with metabolomics data can offer a comprehensive view of the molecular pathways underlying sesame’s nutritional and medicinal properties, potentially opening new avenues for value-added product development. However, sesame cultivation in developing or resource-poor countries may have seldom access to advanced techniques. Hence, international collaborative efforts among institutes, researchers, breeders, and policymakers are essential to address sesame cultivation’s challenges and exploit its full potential as a sustainable oilseed crop. Climate-smart sesame cultivars must be developed to withstand extreme weather events and utilize resources more efficiently to ensure the crop’s sustainability in the face of climate change. Thus, this requires a multidisciplinary approach that combines crossbreeding techniques, genomic tools, and advanced agronomic practices.

## 10. Conclusions

In summary, sesame provides valuable opportunities for oil production and medicinal uses. However, its cultivation is hindered by several factors such as low yields, poor input response, and susceptibility to pests and diseases. Recent advances in molecular breeding provide new ways to overcome these challenges and improve the productivity and profitability of sesame farming. Researchers and breeders can develop superior sesame cultivars by utilizing the extensive genetic diversity found in sesame germplasm and employing advanced breeding techniques. These cultivars are designed to meet growers’ and consumers’ demands worldwide, thus enhancing the crop’s utility and market potential. This comprehensive approach will promote the sustainable growth of the sesame industry, thereby making sesame a more resilient and valuable crop for future generations.

## Figures and Tables

**Table 3:** Transcriptomic profiling of sesame accessions for various stresses.

Transcription Factors	Results	Stress Tolerance	Reference
HD-Zip I-IV	75% of *SiHDz* genes were differentially expressed in response to drought and salinity stress	Drought and salinity	[57]
*SiWRKY*	65 genes were mapped on 15-linkage group	Growth and development Waterlogging and drought stress	[58]
ERF and WRKY	47 genes related to waterlogging resistance	Waterlogging	[59]
*SibZIPs*	Differential expression in response to abiotic stress	Drought, waterlogging, osmotic, salt, and cold stress	[60]
*SindOLP*	Expression under various biotic and abiotic stresses	High expression of ROS scavengers, chlorophyll contents, proline, and low lipid peroxidation	[61]
fIRAK1/4	Expression under drought stress	Peroxidase, heat shock protein, interleukin protein, APETALA2/ethylene-responsive-element-binding protein, and mitogen-activated protein kinase	[62]
GS1-2, PAL, CHS, CAB21,MYB54, MYB88 and NAC75	Three nitrogen levels	Low nitrogen levels affect nitrogen stress tolerance genes through regulator lncRNA MSTRG.13854.1 which affects the MYB54	[42]
MnSOD1, MnSOD2, and PDHA-M	Drought stress	Higher expression of drought-related genes induced through manganese	[63]
SiERFs	Water stress and waterlogging	*SiERF23* and *SiERF54* were induced by both stress conditions	[64]

**Table 5:** Sources of male sterility in sesame.

Male Sterility	Source	Effects	Reference
Genetic male sterility 95ms-5AB	Mutation in Yuzhi 4 by gamma rays Co^60^	Defective pollen, shriveled anthers, recessive gene *Sms1*	[95]
GMS line (W1098A)	Wild accession Yezhi2 (*Sesamum mulayanum* Nair)	Dominant genetic male sterility pollen abortion due to abnormal tapetum	[96]
RGMS (D248A)	Spontaneous male sterility in cultivar Zuzhi	Recessive male sterility. *Ms* gene may be selected by SB2993 and LG1-170	[97]
RGMS	95ms-5A-induced mutant vs. 95ms-5A.	A total of 27 differentially expressed transcripts were identified in sterile vs. fertile buds. Eleven transcripts were involved in energy metabolism, signal transduction, and cell development	[98]
CMS	*Sesamum malabaricum*	Interspecific hybridization showed male sterility. Reciprocal effects were identified indicating cytoplasmic inheritance of organelles	[99]
